# Altered Parietal Activation during Non-symbolic Number Comparison in Children with Prenatal Alcohol Exposure

**DOI:** 10.3389/fnhum.2017.00627

**Published:** 2018-01-08

**Authors:** Keri J. Woods, Sandra W. Jacobson, Christopher D. Molteno, Joseph L. Jacobson, Ernesta M. Meintjes

**Affiliations:** ^1^Division of Biomedical Engineering, Department of Human Biology, University of Cape Town, Cape Town, South Africa; ^2^Department of Human Biology, Faculty of Health Sciences, University of Cape Town, Cape Town, South Africa; ^3^Department of Psychiatry and Behavioral Neurosciences, Wayne State University School of Medicine, Detroit, MI, United States; ^4^Department of Psychiatry and Mental Health, Faculty of Health Sciences, University of Cape Town, Cape Town, South Africa

**Keywords:** arithmetic deficits, fetal alcohol spectrum disorders, fetal alcohol syndrome, fMRI, non-symbolic number processing, parietal lobe, intraparietal sulci, numerical distance effect

## Abstract

Number processing is a cognitive domain particularly sensitive to prenatal alcohol exposure, which relies on intact parietal functioning. Alcohol-related alterations in brain activation have been found in the parietal lobe during symbolic number processing. However, the effects of prenatal alcohol exposure on the neural correlates of non-symbolic number comparison and the numerical distance effect have not been investigated. Using functional magnetic resonance imaging (fMRI), we examined differences in brain activation associated with prenatal alcohol exposure in five parietal regions involved in number processing during a non-symbolic number comparison task with varying degrees of difficulty. fMRI results are presented for 27 Cape Colored children (6 fetal alcohol syndome (FAS)/partial FAS, 5 heavily exposed (HE) non-sydromal, 16 controls; mean age ± SD = 11.7 ± 1.1 years). Fetal alcohol exposure was assessed by interviewing mothers using a timeline follow-back approach. Separate subject analyses were performed in each of five regions of interest, bilateral horizontal intraparietal sulci (IPS), bilateral posterior superior parietal lobules (PSPL), and left angular gyrus (left AG), using the general linear model with predictors for number comparison and difficulty level. Mean percent signal change for each predictor was extracted for each subject for each region to examine group differences and associations with continuous measures of alcohol exposure. Although groups did not differ in performance, controls activated the right PSPL more during non-symbolic number comparison than exposed children, but this was not significant after controlling for maternal smoking, and the right IPS more than children with fetal alcohol syndrome (FAS) or partial FAS. More heavily exposed children recruited the left AG to a greater extent as task difficulty increased, possibly to compensate, in part, for impairments in function in the PSPL and IPS. Notably, in non-syndromal heavily exposed children activation was impaired in the right PSPL, but spared in the right IPS. These results extend previous findings of poor right IPS recruitment during symbolic number processing in FAS/PFAS, indicating that mental representation of relative quantity is affected by prenatal alcohol exposure for both symbolic and non-symbolic representations of quantity.

## Introduction

Prenatal alcohol exposure causes impairment in brain structure and function, leading to cognitive and behavioral deficits (Archibald et al., 2001; Sowell et al., 2001; Riley and McGee, 2005; Astley et al., 2009). The alcohol-related cognitive deficits include lower IQ (Streissguth et al., 1990; Jacobson et al., 2004), poor attention, and executive function (Kodituwakku et al., 1995; Coles et al., 1997; Mattson et al., 1999; Burden et al., 2005b), and slower cognitive processing speed (Streissguth et al., 1990; Jacobson et al., 1993, 1994; Coles et al., 2002). Among the cognitive deficits seen in relation to prenatal alcohol exposure, arithmetic has been found to be especially sensitive, and mathematical deficits persist, even after controlling for IQ (Streissguth et al., 1990, 1994a; Coles et al., 1991; Chiodo et al., 2004; Jacobson et al., 2004; Burden et al., 2005a). When academic achievement tests are administered to alcohol-exposed individuals, arithmetic is consistently more impaired than reading or spelling (Streissguth et al., 1991; Goldschmidt et al., 1996; Kerns et al., 1997; Howell et al., 2006). Moreover, alcohol-related deficits in numerosity can already be detected in infancy (Jacobson et al., 2011b).

Fetal alcohol syndrome (FAS), the most severe of the fetal alcohol spectrum disorders (FASD), is characterized by distinctive craniofacial dysmorphology [short palpebral fissures, thin upper lip (vermillion), flat philtrum], small head circumference and pre- and/or postnatal growth retardation (Hoyme et al., 2005). In partial FAS (PFAS), some of the craniofacial dysmorphology is seen, as well as small head circumference, retarded growth, or neurobehavioral deficits. Heavily exposed (HE) individuals lacking the distinctive dysmorphology are diagnosed with alcohol related neurodevelopmental disorder (ARND) if they exhibit cognitive and/or behavioral impairment (Stratton et al., 1996; Hoyme et al., 2005).

Since the beginning of the 20th century, studies have shown that the parietal lobe is involved in number processing (Henschen, 1919), but more recently functional magnetic resonance imaging (fMRI) has provided a more extensive understanding of the neuroanatomy of this domain of processing. Based on brain lesion and neuroimaging findings, Dehaene (1992) and Dehaene and Cohen (1996) have proposed a triple-code model of number processing that incorporates three different systems of representation: the quantity system, the verbal system and the visual system. They have posited that the core quantity system—a non-verbal abstract representation of numerical quantity—is localized bilaterally in the anterior portion of the horizontal segment of the intraparietal sulci (IPS). This area is hypothesized to support number processing irrespective of the notation used, that is, whether represented symbolically as Arabic numbers or sequences of words or non-symbolically by, for example, numbers of dots. Verbal processing of numbers is posited to be based in the left angular gyrus (left AG; close to the language areas of the brain), while the bilateral posterior superior parietal lobules (PSPL) are hypothesized to be involved in spatial and non-spatial attentional processes contributing to the visual processing of quantity.

Magnitude comparison can be studied by asking the subject to determine which of two numbers is larger or in a proximity judgment (PJ) paradigm (“Which of two numbers is closer to a third?”). Behavioral studies of magnitude comparison have shown that reaction time is slower for comparison of numbers that are closer together (e.g., 2 and 3) than numbers that are farther apart (e.g., 2 and 9), a phenomenon referred to as the “distance effect” (Moyer and Landauer, 1967). Consistent with this behavioral pattern, studies have shown that the IPS is more active when comparing numbers that are closer together (Pinel et al., 2001; Kaufmann et al., 2005; Ansari and Dhital, 2006; Mussolin et al., 2010a).

In the first fMRI study of number processing in FASD, adults with and without prenatal alcohol exposure were administered a subtraction task (Santhanam et al., 2009). Exposed individuals with alcohol-related dysmorphology exhibited poorer task performance and lower activation in parietal and frontal regions known to be associated with arithmetic processing, including the right inferior and left superior parietal regions and medial frontal gyrus, compared with controls. In the first fMRI study of children with FASD, the participants were administered tasks involving PJ and single-digit addition problems, which they performed as well as controls because the tasks had been simplified for administration in the scanner (Meintjes et al., 2010). Children with FAS and PFAS activated a markedly more diffuse parietal region extending into the precuneus and posterior cingulate, and for exact addition (EA), also into the postcentral gyrus. The FAS/PFAS group also exhibited significantly greater activation of the left AG than the control children in the PJ task. However, no significant differences were detected in the anterior portion of the IPS and other regions which have been linked by Dehaene et al. (2003) and associates to number processing, possibly due to lack of statistical power in the small sample on which this whole brain voxel-wise analysis was conducted. Using a region of interest (ROI) analysis, we recently examined brain activation patterns in the five parietal regions identified by Dehaene et al. (2003) as most critical for number processing in a sample consisting of the FAS/PFAS and control children from the Meintjes et al. (2010) study and a group of non-syndromal HE children, which was assessed on both the PJ and EA tasks (Woods et al., 2015). During both tasks, higher levels of prenatal alcohol exposure were associated with weaker activation of the right IPS. Additionally, during PJ, children in the FAS/PFAS group activated the left AG more than control or HE children.

These and other studies examining the effects of prenatal alcohol exposure on neural correlates of number processing have, however, focused on symbolic representation of quantity (e.g., using Arabic numbers or written number words) (Streissguth et al., 1994a,b; Kopera-Frye et al., 1996; Burden et al., 2005a; Santhanam et al., 2009; Meintjes et al., 2010; Jacobson et al., 2011a; Woods et al., 2015). To date, no neuroimaging studies have examined non-symbolic number processing (e.g., dot patterns or collections of objects) in alcohol exposed children. Though symbolic representation of number appears to be localized in the IPS in adults (Dehaene et al., 2003), a meta-analysis of studies comparing number processing in children vs. adults suggests that the location of parietal activations is more notation specific in children (Kaufmann et al., 2011). One aim of the current study is to examine the degree to which the effects of prenatal alcohol exposure on symbolic number processing are also seen in non-symbolic number processing, which also depends heavily on the core quantity system.

Studies of other pediatric conditions associated with mathematical difficulties, such as developmental dyscalculia (DD; characterized by impairment in the processing of numerical and arithmetical information in individuals with normal intelligence), have shown a more pronounced behavioral distance effect in affected children than typically developing controls (Price et al., 2007; Mussolin et al., 2010b), and poorer math achievement has also been associated with a greater distance effect (Gullick et al., 2011). Similarly, higher levels of prenatal alcohol exposure were found to be associated with a more pronounced distance effect in a behavioral study using a Sternberg reaction time paradigm (Burden et al., 2005a). In DD, activations of the bilateral IPS fail to exhibit the increased response to differences in numerical distance seen in normal control children (Mussolin et al., 2010a).

In this study, we used fMRI to investigate the effect of prenatal alcohol exposure on the neural correlates of the numerical distance effect during a non-symbolic number comparison task. To our knowledge, the effect of prenatal alcohol exposure on the neural correlates of the distance effect and non-symbolic number comparison has not to date been investigated. Based on studies of DD (Price et al., 2007; Mussolin et al., 2010a) and our previous math studies (Meintjes et al., 2010; Woods et al., 2015), we hypothesized that prenatal alcohol exposure would be associated with weaker activation in the right IPS during non-symbolic number comparison, as well as weaker increases in activation in the right IPS arising from the distance effect. Based on our previous studies, we also expected to find increased compensatory activation in the left AG by children with FASD.

## Materials and Methods

### Participants

Participants were right-handed children from the Cape Colored (mixed ancestry) community in Cape Town, South Africa. The Cape Colored community is composed primarily of descendants of white European settlers, Malaysian slaves, Khoi-San aboriginals, and black African ancestors. The incidence of FASD in this population is exceptionally high due to poor socioeconomic circumstances and historical practices of compensating farm labor with wine, which have contributed to a tradition of heavy recreational weekend binge drinking (May et al., 2007, 2013).

Data from 7 of 34 children recruited into the study were excluded from the analyses: technical error (1 control child), incomplete imaging data (2 controls), functional data due to failure to meet the performance criteria (1 FAS, 1 control), and excessive motion (1 FAS, 1 control). Mean and maximum displacements did not differ between exposure groups [*t*(25) = 1.49, *p* = 0.162 and *t*(25) = 1.55, *p* = 0.148, respectively].

After exclusions, there were 27 children with usable data (11 alcohol exposed and 16 controls). The children with usable scanner data did not differ from those excluded in terms of alcohol exposure or FASD diagnostic group (all *p*s > 0.20) or other demographic variables, including child age, sex, IQ, and blood lead concentration, and maternal education, age at delivery, parity, and smoking during pregnancy (all *p*s > 0.10).

13 of the 27 children were the older siblings of participants in our Cape Town Longitudinal Cohort (Jacobson et al., 2008). The others were identified by screening all of the 8- to 12-year-old children from an elementary school in a rural section of Cape Town, where there is a very high incidence of alcohol abuse among local farm workers (Meintjes et al., 2010; Jacobson et al., 2011c).

### Procedure

A research nurse and staff driver transported the mother and child from their home to the Cape Universities Brain Imaging Centre (CUBIC). All examiners were blind with regard to maternal alcohol history and the child’s FASD diagnostic status, except in the most severe cases. Written informed consent was obtained from each mother and assent from each child. Approval for human research was obtained from the ethics committees at Wayne State University and the UCT Faculty of Health Sciences.

Each mother was interviewed in her primary language, Afrikaans or English, regarding her alcohol consumption and smoking during pregnancy. The alcohol interviews used a timeline follow-back approach (Sokol et al., 1985; Jacobson et al., 2002) to determine incidence and amount of drinking on a day-by-day basis during pregnancy. By contrast to most of our Cape Town studies, in which the mothers were interviewed prospectively during pregnancy, the interviews for this sample were conducted retrospectively. Any child whose mother reported consuming at least 14 standard drinks/week (1.0 oz absolute alcohol (AA)/day) on average or engaged in binge drinking during pregnancy (4 or more drinks/occasion) was considered heavily exposed. Controls were children whose mothers reported abstaining or drinking less than seven drinks/week and no binge drinking during pregnancy. Volumes recorded for each type of beverage consumed each day were converted to oz AA using multipliers proposed by Bowman et al. (1975) to provide three continuous measures of drinking during pregnancy: average oz AA/day, AA/drinking day (dose/occasion) and frequency (days/wk). Number of cigarettes smoked on a daily basis was also recorded; use of illicit drugs was obtained as days/month. Mothers were also interviewed regarding their education and occupational status and that of their spouse/partner to determine socioeconomic status (SES) on the Hollingshead (2011) scale. Mothers and children were given breakfast, lunch, and a snack at each laboratory visit. The mother received a small monetary compensation for each visit, as well as a photograph of her child, and the child was given a small gift.

Each child was examined for growth and FAS dysmorphology by two expert United States-based dysmorphologists during a clinic conducted in 2005 (Jacobson et al., 2011c) using a standard protocol based on the Revised Institute of Medicine criteria (Hoyme et al., 2005). The criteria for full FAS were at least two of the principal dysmorphic features, small head circumference (bottom 10th percentile), and low weight or short stature (bottom 10th percentile); for partial FAS (PFAS), two features and small head circumference, low weight, or short stature. Alcohol-exposed children who did not meet criteria for either FAS or PFAS were designated non-syndromal HE.

There was substantial agreement among the examiners on the assessment of all dysmorphic features, including the three-principal fetal alcohol related features—philtrum and vermilion measured on the Astley and Clarren (2001) rating scales and palpebral fissure length (median *r* = 0.78). Two children who could not attend the 2005 clinic were examined by a Cape Town based FASD expert dysmorphologist (N. Khaole, MD, United States), whose diagnoses were subsequently confirmed by examinations conducted in follow-up clinics we held with the same dysmorphologists in 2009 and with HEH in 2013 and 2016.

### Neuropsychological Assessment

Each child was assessed on 7 of the 10 subtests from the Wechsler Intelligence Scale for Children, Third Edition (WISC-III)—Similarities, Arithmetic, Digit Span, Symbol Search, Coding, Block Design, and Picture Completion—and Matrix Reasoning from the WISC-IV. The IQ subtests were selected to represent the four dimensions of the WISC-III: Verbal Comprehension (Similarities), Perceptual Organization (Block Design, Picture Completion, Matrix Reasoning), Freedom from Distractibility (Digit Span, Arithmetic), and Processing Speed (Coding, Symbol Search). Similarities was the only subtest administered in the verbal domain to limit use of tests that may be more dependent on the cross-cultural context. IQ was estimated from these subtests using Sattler’s (1992) formula for computing Short Form IQ; validity coefficients for Short Form IQ based on 5 or more subtests consistently exceed *r* = 0.90. Handedness was assessed on the Annett (1970a,b) Behavioral Handedness Inventory.

### Neuroimaging Assessment

#### Magnetic Resonance Imaging Protocol

All scans were acquired using a 3T Allegra MRI scanner (Siemens Medical Systems, Erlangen, Germany). High-resolution anatomical images were acquired in sagittal orientation using a magnetization prepared rapid gradient echo (MPRAGE) sequence (TR 2300 ms, TE 3.93 ms, TI 1100 ms, 160 slices, flip angle 12°, 1.3 mm × 1.0 mm × 1.0 mm, 6.03 min). During the fMRI protocol, 126 functional volumes sensitive to blood oxygen level dependent contrast were acquired with a T2^∗^-weighted gradient echo, echo planar imaging sequence (TR = 2000 ms, TE = 30 ms, 34 interleaved slices, 3 mm thick, gap 0.9 mm, 200 mm field of view, resolution 3.125 mm × 3.125 mm × 3 mm, 4.2 min). Total scan time was less than 1 hour since these tasks formed part of a longer protocol.

#### Functional MRI Experimental Task

**FIGURE 1:**
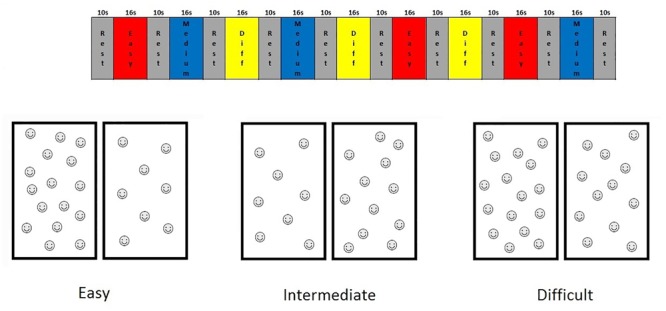
Timing diagram of the Smarties task and examples of the stimuli for each difficulty level.

For this study, we designed an fMRI non-symbolic number comparison task, which we refer to here as the “smarties” test. For this test, the child is presented with a screen split vertically in half, each half containing different numbers of smiley faces, and is asked to press the button on the side with the most smiley faces. This non-symbolic number comparison task was administered using a fixed-paced block design and had nine 16-s task blocks, each with eight problems, interleaved with 10-s rest blocks. Each task block comprised problems at 1 of 3 levels of difficulty, defined in terms of the ratio of number of faces on one side of the screen to the other (1:2, 2:3, 3:4) (see **Figure 1**). Stimuli were shown for only 1 s to prevent counting. During rest blocks, the child looked at a fixation square.

Each child practiced this task initially in a mock scanner to reduce anxiety, thereby facilitating completion of the MRI scans. The experimental task was programmed using E-Prime software (Psychology Software Tools, Inc., Pittsburgh, PA, United States) and was presented on a rear projection screen using a data projector located in a room behind the scanner through a waveguide in-line with the bore of the magnet. The child held a Lumitouch response system (Photon Control Inc., Burnaby, CO, Canada) in his/her right hand and responded using the right index and middle finger. The child was able to talk to the examiner using an intercom built into the scanner and could stop the scan at any time by squeezing a ball held in his/her left hand.

#### Behavioral Performance

Behavioral responses were recorded on a computer; number of problems correct and reaction time for correct responses were tabulated. Blocks with fewer than 5 correct responses were considered bad blocks. If 5 or more of a subject’s nine active blocks were bad, their data were excluded (1 exposed, 1 control). All children met the performance criterion of at least one usable block at each difficulty level.

#### fMRI Analysis

All fMRI analyses were performed using Brain Voyager QX (Brain Innovation, Maastricht, Netherlands). Four dummy images were acquired in each run that were excluded from all analyses. Images were motion corrected relative to its first volume with trilinear/sinc interpolation. Images were corrected for different slice acquisition times and linear trends and temporally smoothed with a high pass filter of 2 cycles/point. For each child, data from the largest section with no movement greater than 3 mm displacement or 3.0° rotation were analyzed. Children were excluded from further analyses if the section of usable data did not include at least one block from each condition, resulting in two additional children being excluded (1 exposed, 1 control). Each child’s functional data were co-registered to his/her high-resolution anatomical MRI, rotated into the AC-PC plane and normalized to Talairach space using a linear transform calculated on the anatomical images. The 3.125 mm × 3.125 mm × 3 mm fMRI voxels were interpolated during Talairach normalization to 3 mm × 3 mm × 3 mm.

**FIGURE 2:**
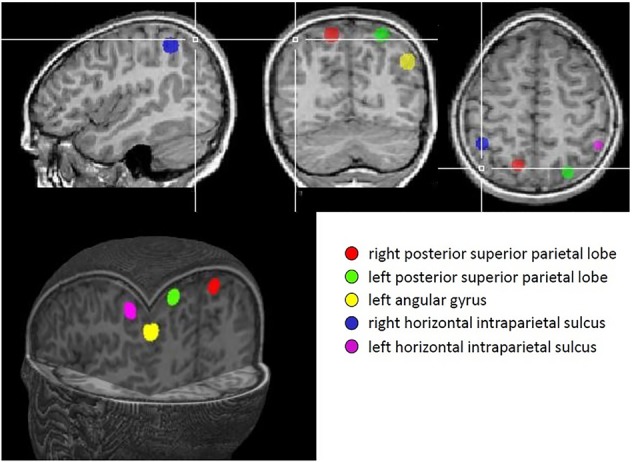
Regions identified in Dehaene’s meta-analysis that were used as regions of interest in this study.

*A priori* regions of interest (ROIs) were defined for each of the five parietal regions identified in Dehaene et al.’s (2003) meta-analysis; namely, bilateral anterior horizontal IPS, bilateral PSPL and left AG. Each ROI consisted of a sphere, radius 6 mm, centered on the coordinates derived from the meta-analysis. These regions are illustrated in **Figure 2**.

To create a parametric model, difficulty levels were weighted by the ratio of the number of faces on the two sides of the stimuli within each block. We used two predictors of interest. The first predictor (“main”) gives the signal variation explained by non-symbolic number processing across all task difficulties. The other predictor (“parametric”) gives the parametric increase in activation as the difficulty level increases and is a measure of the strength of the distance effect. Separate subject analyses were performed on the average signal in each ROI using the general linear model with predictors of interest convolved by the standard hemodynamic function. The six motion correction parameters were *z*-transformed and then added as predictors of no interest. For each predictor of interest, beta maps estimating mean percent signal change were created for each subject for each ROI.

#### Statistical Analyses

All variables were examined for normality of distribution. AA/day was skewed (skewness > 3) and was log transformed (log X + 1). The following variables with outliers greater than 3 SDs beyond the mean were transformed by recoding all outlying values to one point beyond the next most extreme observed value: maternal education (*n* = 1), AA/occasion (*n* = 1), and proportional drinking days (*n* = 1).

Seven control variables were assessed for consideration as potential confounders of the relation of prenatal alcohol exposure to number processing: four demographic characteristics (parity, SES, mother’s age at delivery, and years of education), two child characteristics (child sex and age at assessment), and two exposure variables (number of cigarettes smoked per day during pregnancy and level of postnatal lead exposure). Lead exposure, which was based on a venous blood sample obtained from the child, was included because lead levels in this population are within the range in which subtle but meaningful effects on cognitive function have consistently been reported (e.g., Lanphear et al., 2000; Chiodo et al., 2004). Each control variable that was even weakly related to a given outcome measure (at *p* < 0.10) was considered a potential confounder of the effect of alcohol exposure on the outcome in question.

*T*-tests were used to examine differences between diagnostic groups (exposed; control) on the behavioral and neuroimaging outcome measures. Analysis of covariance (ANCOVA) was used to test whether differences remained significant after controlling for potential confounders. Differences in performance between difficulty levels were examined using a repeated measures ANOVA with Greenhouse–Geisser correction. The relation of the three continuous measures of prenatal alcohol exposure—AA/day, AA/drinking day and proportional drinking days—to each of the outcome measures was examined using Pearson correlation. Multiple regression analyses were then run relating each of the continuous exposure measures and potential confounders to each of the outcomes.

Association of percent signal change in each of the ROIs to behavioral performance was examined using Pearson correlation.

## Results

### Sample Characteristics

The sample characteristics are summarized in **Table 1**. The mothers of the alcohol exposed children reported an average of 13.4 standard drinks of alcohol per drinking occasion during pregnancy on 2–3 days per week. By contrast, all but two women in the control group abstained from drinking during pregnancy; the one control mother drank two drinks once during pregnancy and the other control mother drank two drinks about once a month. The groups were generally similar in terms of the other background characteristics, except that mothers of the exposed children smoked more during pregnancy than mothers of controls. None of the mothers in this sample reported using drugs during pregnancy. Of the 11 alcohol-exposed children, 6 met criteria for either FAS or PFAS, while 5 were designated as non-syndromal HE. As expected, the IQ scores of the exposed children were lower than those of the controls. The low IQ scores of both groups reflect the highly disadvantaged circumstances and poor quality of education available in this community.

**Table 1:** Sample characteristics.

	Exposed (*n* = 11)	Control (*n* = 16)	*t* or χ^2^
**Prenatal alcohol exposure**			
Absolute alcohol (AA)/day (oz)	2.8 (2.5)	0.0 (0.0)	3.73^∗∗^
AA/occasion (oz)	6.7 (3.6)	0.1 (0.4)	6.06^∗∗∗^
Frequency (days/week)	2.8 (1.4)	0.0 (0.0)	12.49^∗∗∗^
**Potential confounders**			
*Maternal*			
Parity	2.1 (1.0)	2.8 (1.5)	1.27
Years of education	7.2 (2.8)	8.6 (1.8)	1.65
Smoking during pregnancy (cigs/day)	7.5 (7.8)	1.9 (3.6)	2.48^∗^
Mother’s age at delivery	25.0 (4.2)	27.6 (4.5)	1.48
*Child*			
Sex (% male)	18.2	37.5	1.17
Age at assessment	11.8 (1.0)	11.6 (1.2)	0.48
Blood lead concentration (μg/dl)^a^	6.4 (2.3)	5.9 (2.1)	0.58
**Potential mediators**			
Estimated IQ score	61.8 (9.8)	74.7 (11.3)	3.07^∗∗^

*Means (SD); ^†^*p* < 0.10, ^∗^*p* < 0.05, ^∗∗^*p* < 0.01, ^∗∗∗^*p* < 0.001; ^a^Data only available for 25 subjects.*

### Neuropsychological Assessments

Behavioral data are summarized in **Table 2**. Overall accuracy and reaction time did not differ between exposed and control children (all *p*s > 0.30), nor did accuracy and reaction time at each difficulty level (all *p*s > 0.25). None of the behavioral measures were related to the continuous measures of prenatal alcohol exposure, although the relation between reaction time in the easy level of the task and AA/occasion fell just short of significance (*r* = 0.34, *p* < 0.010), with greater alcohol exposure associated with slower reaction time, as expected.

**Table 2:** Comparison of behavioral performance by exposure group.

	Exposed	Control	*p*
	(*n* = 11)	(*n* = 16)	
**Accuracy (% correct)**			
Easy	87.9 (9.9)	89.1 (10.0)	0.764
Intermediate	86.7 (11.0)	90.6 (9.1)	0.325
Difficult	79.9 (11.0)	79.9 (11.0)	0.425
Overall	84.8 (8.8)	87.8 (8.4)	0.394
**RT (ms)**			
Easy	693.1 (76.5)	669.3 (80.2)	0.448
Intermediate	739.7 (79.9)	706.9 (74.9)	0.286
Difficult	759.5 (113.5)	735.1 (87.5)	0.534
Overall	727.7 (78.1)	701.2 (65.3)	0.348

*Values are Means (SD).*

Performance accuracy was high in both groups, with scores over 80% for all but the most difficult level. A repeated measures ANOVA with Greenhouse–Geisser correction showed that mean accuracy differed significantly between levels [*F*(1.9,48.7) = 7.60, *p* = 0.002], with greater accuracy in the easy and intermediate levels than in the difficult level (*p* = 0.020 and *p* = 0.005, respectively). Mean reaction times also differed between difficulty levels [*F*(1.9,49.6) = 9.89, *p* < 0.0005], with reaction times in the easy condition being faster than in the intermediate (*p* < 0.013) and difficult (*p* < 0.001) conditions.

### Neuroimaging Assessments

**Table 3** shows mean % signal change during non-symbolic number comparison in the Dehaene parietal ROIs for children in each of the two groups. The only significant group difference was in the right PSPL, where the control group showed greater activation than the exposed group, in which activations were actually reduced lower than baseline, on average, however, this effect was no longer significant after adjustment for smoking during pregnancy. By contrast, the left AG showed a stronger distance effect in the exposed children than in the controls (see **Table 4**).

**Table 3:** Mean percent signal change in the *a priori* regions of interest during number comparison (across all difficulty levels).

	Talairach coordinates	Exposed *(n* = 11)	Control (*n* = 16)	*F*_1_	*F*_2_
R posterior superior parietal lobule^a^	15,–63,56	–0.2 (0.4)	0.1 (0.4)	4.67^∗^	0.43
L posterior superior parietal lobule^a,b,c^	–22,–68,56	0.1 (0.5)	0.3 (0.8)	0.61	1.76
L angular gyrus^d^	–41,–66,36	–0.1 (0.3)	–0.2 (0.2)	0.40	0.24
R intraparietal sulcus^c^	41,–47,48	0.0 (0.3)	0.1 (0.3)	1.66	0.80
L intraparietal sulcus^b^	–44,–48,47	0.0 (0.4)	0.1 (0.2)	0.06	0.90

*F_*1*_= before adjustment for potential confounders; *F*_*2*_= after adjustment for potential confounders; ^∗^*p* < 0.05; d.f. = (1,25); ^a^adjusted for smoking during pregnancy; ^b^adjusted for maternal education; ^c^adjusted for child’s sex; ^d^adjusted for child’s age.*

**Table 4:** Mean parametric increase in activation with increasing task difficulty in the *a priori* regions of interest (parametric effect).

	Talairach coordinates	Exposed *(n* = 11)	Control (*n* = 16)	*F*_1_	*F*_2_
R posterior superior parietal lobule	15,–63,56	–1.0 (1.9)	–0.5 (2.6)	0.24	0.24
L posterior superior parietal lobule	–22,–68,56	–2.4 (3.0)	–1.8 (5.0)	0.15	0.15
L angular gyrus	–41,–66,36	0.7 (1.1)	–0.8 (1.9)	5.86^∗^	5.87^∗^
R intraparietal sulcus^a,b^	41,–47,48	0.0 (1.3)	–0.2 (1.2)	0.13	1.33
L intraparietal sulcus	–44,–48,47	0.2 (1.0)	–0.7 (2.1)	1.88	1.46

**F*_*1*_ = before adjustment for potential confounders; *F*_*2*_ = after adjustment for potential confounders; ^∗^*p* < 0.05; d.f. = (1, 25); ^a^controlled for maternal education; ^b^controlled for smoking during pregnancy.*

Since we did not observe a difference in activation during number comparison between exposed and control children in the right IPS as hypothesized, we compared activation levels in this region in the FAS/PFAS group only to the controls. As predicted, the control children activated the right IPS more than the FAS/PFAS group [*t*(20) = 2.14, *p* = 0.045, means ± SD = –0.15 ± 0.12 and 0.11 ± 0.28 for the FAS/PFAS and control groups, respectively]. When the FAS/PFAS, non-syndromal HE, and control children were compared, activation patterns in the right PSPL were similar for the FAS/PFAS and HE groups [*t*(9) = 1.21, *p* = 0.257], whereas activation patterns in the right IPS of HE children were more similar to those of controls [*t*(19) = 0.021, *p* = 0.986].

**FIGURE 3:**
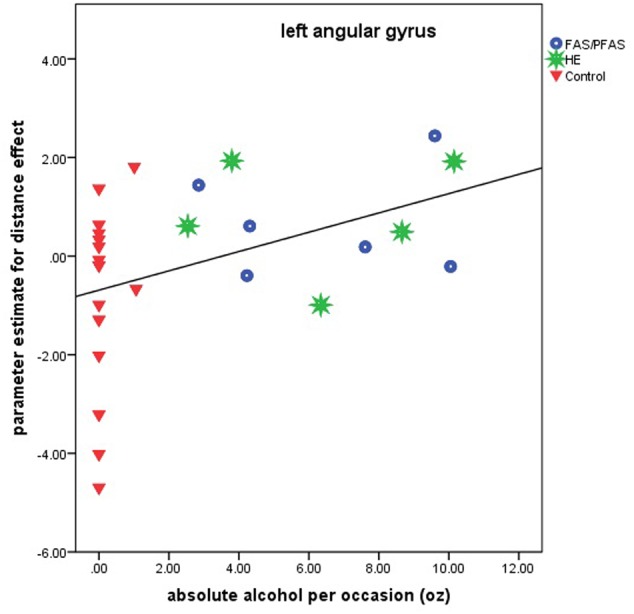
Relation of the parameter estimate for the distance effect in the left angular gyrus to absolute alcohol (AA) per occasion (6 children with FAS/PFAS, 5 heavily exposed non-syndromal (HE), 16 control children).

**FIGURE 4:**
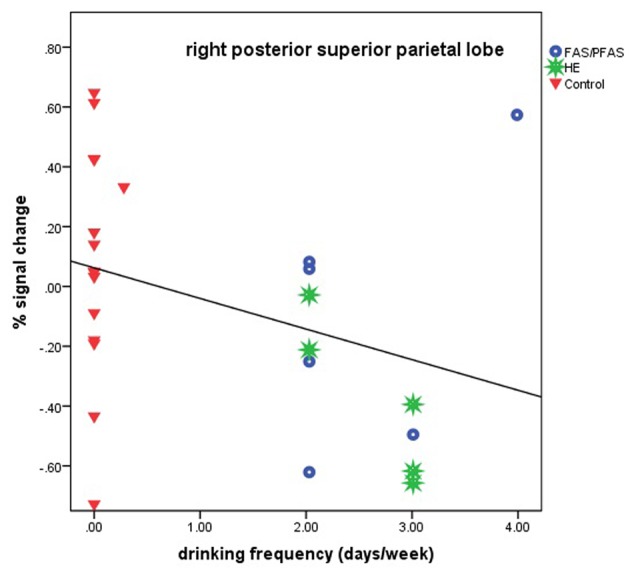
Relation of percent signal change in the right posterior superior parietal lobule to frequency of drinking (6 children with FAS/PFAS, 5 heavily exposed non-syndromal (HE), 16 control children).

Higher levels of prenatal alcohol exposure on all three continuous measures were related to greater distance effects in the left AG (*r*s = 0.46, 0.41, and 0.45 for AA/day, AA/occasion and frequency of drinking, respectively, all *p*s < 0.05; **Figure 3**). More drinking days/week was related to reduced activation during number comparison in the right PSPL (*r* = –0.41, *p* = 0.036; **Figure 4**).

Children who showed a greater distance effect in the left PSPL (i.e., activated the left PSPL more with increasing task difficulty) had shorter reaction times (*r* = –0.43, *p* = 0.03) and those who showed greater activation of this region had better accuracy (*r* = 0.46, *p* = 0.02).

## Discussion

This study examined the relation of FASD diagnosis and continuous measures of prenatal alcohol exposure to activation of five parietal regions, which have been identified by Dehaene et al. (2003) as most critical for number processing, during a non-symbolic number comparison task with varying degrees of difficulty. Despite similar behavioral performance, prenatal alcohol exposure was associated with altered patterns of brain activation. Control children showed greater activation of the right PSPL during non-symbolic number comparison than exposed children; however, this effect was no longer significant after adjusting for smoking. Control children also showed greater activation of the right IPS than children with a diagnosis of FAS or PFAS. Both greater activation of the left PSPL and a greater distance effect on the activation of this region were associated with better task performance. In the right PSPL, the activation patterns of HE children were similar to those of the FAS/PFAS group, while in the right IPS their activation patterns were similar to that of the controls. With respect to the distance effect, the exposed children showed greater activation with increasing levels of task difficulty in the left AG, compared to the controls.

### Effects on the IPS

As predicted, prenatal alcohol exposure was associated with weaker activation of the right IPS during non-symbolic number comparison, although the effect was seen only in the syndromal children in this sample. This finding of an adverse effect of prenatal alcohol exposure on activation of the right IPS during non-symbolic number processing is consistent with previous studies of symbolic number processing (Santhanam et al., 2009; Meintjes et al., 2010; Woods et al., 2015). During a subtraction task (Santhanam et al., 2009), adults with alcohol related dysmorphology showed weaker activation of the right inferior parietal lobe (just slightly superior and posterior to our right IPS ROI) than unexposed controls. This effect was not seen in the non-dysmorphic prenatal alcohol exposure group. In a whole brain analysis (Meintjes et al., 2010), we found that control children activated the right IPS more than children with FAS/PFAS during a PJ task, though this region was centered more posteriorly than the right IPS ROI used in this study. There were no non-dysmorphic HE children included in that study. In our previous study of symbolic number processing using the same *a priori* ROIs as in the present study, we found that increasing alcohol exposure was related to weaker activation in the right IPS during both PJ and simple addition (Woods et al., 2015). These results, taken together with the findings from the current study, indicate that prenatal alcohol exposure impairs the activation of the right IPS during both symbolic and non-symbolic number processing.

The bilateral IPS have frequently been linked to non-verbal representation of quantity (Dehaene et al., 2003). This region is activated when numbers are attended to, even without any explicit number processing task requirements (Eger et al., 2003), during number comparison (Chochon et al., 1999), and during mental arithmetic (Burbaud et al., 1999; Chochon et al., 1999; Pesenti et al., 2000). The region is independent of modality of number and is activated whether number is presented as Arabic numbers, written number words, spoken numbers or sets of dots (Le Clec’H et al., 2000; Pinel et al., 2001; Piazza et al., 2002). It is more active when manipulating large numbers (Kiefer and Dehaene, 1997; Stanescu-Cosson et al., 2000), performing arithmetic with three rather than two operands (Rivera et al., 2002), approximating addition rather than computing the exact sum (Dehaene et al., 1999), and performing subtraction rather than multiplication (Chochon et al., 1999; Lee, 2000). This region has also been shown to exhibit a distance effect, meaning that it is activated more when comparing numbers that are closer together (Dehaene and Cohen, 1996; Pinel et al., 2001). Although we did not observe a behavioral difference in this fMRI study, evidence from our Detroit and Cape Town behavioral studies suggests a specific effect of prenatal alcohol on magnitude comparison (Burden et al., 2005a; Jacobson et al., 2011a,b). The poorer recruitment of the IPS in children with FAS or PFAS observed here provides further evidence of a fetal alcohol exposure related change in mental representation and manipulation of quantity.

The non-dysmorphic HE children did not show poorer recruitment on the IPS than control children. Given that some of the non-dysmorphic children were very heavily exposed, perhaps the effect of prenatal alcohol on the right IPS relates more to the timing of drinking during pregnancy than on the quantity the mother drank.

White matter microstructure directly underlying the left IPS has been shown to be related to mathematical abilities in children with prenatal alcohol exposure. Higher math scores were associated with greater fractional anisotropy in this region (Lebel et al., 2010). These findings, combined with our results and the results of previous fMRI studies, suggest that both gray and white matter of the IPS are involved in mathematical processing and that both are affected by prenatal alcohol exposure.

Based on the findings from developmental dyscalculia (Price et al., 2007; Mussolin et al., 2010a), we expected to find a weaker distance effect in the right IPS in children with prenatal alcohol exposure; however, this was not the case. Although the control children activated the right IPS more than the FAS/PFAS group during non-symbolic number comparison averaged across the three difficulty levels, there was no difference in the distance effect between the groups in this region. The lack of a group difference in the distance effect in this region may be related to the fact that the distance effect was not clearly evident in the right IPS in the control children, possibly because they were too young and their brains still too immature. In a symbolic number comparison task, adults showed a distance effect in parietal regions, including the intraparietal sulcus (Kaufmann et al., 2005), while in an identical paradigm, 8- to 12-year-old children showed no significant distance effect in any parietal region, even though they showed a behavioral distance effect (Kaufmann et al., 2006). An alternative reason may relate to task design. Our “smiley face” stimuli remained the same size throughout trials, so it is possible that children used non-numerical cues, such as density of the faces, or the total area covered by the faces to select the correct answer. Other studies have varied the sizes of their stimuli, so that the participants could not use these non-numerical cues to select the correct answer (Ansari et al., 2006; Holloway and Ansari, 2009; Gullick et al., 2011; Kucian et al., 2011; Lonnemann et al., 2011). A third possibility is that the task did not increase enough in difficulty for the additional intraparietal neuronal resources to be required. However, this is unlikely, as the behavioral results did show distance effects.

### Effects on the Left AG

Consistent with our hypothesis, exposed children showed compensatory activation of the left AG, demonstrating a greater distance effect in that region than typically developing controls. This finding was also evidenced by an association between level of prenatal alcohol exposure and the distance effect in the left AG. These results suggest that alcohol exposed children need to recruit the left AG to a greater extent as the task difficulty increases, possibly to compensate for deficits in quantity representation in the IPS. In contrast, controls showed an inverse distance effect in this region, that is, reduced left AG activation during the more difficult conditions, presumably due to better functioning of regions specialized for quantity representation. This area has also been implicated in our previous studies of number processing in FASD. In a whole brain voxelwise analysis, we found a significant group difference in a nearly identical region of the left AG [–42,–65,36] with greater activation in the FAS/PFAS group than the controls (Meintjes et al., 2010), and in an ROI study, we found that during PJ children with FAS or PFAS demonstrated greater activation of the left AG ROI than HE or control children (Woods et al., 2015).

The AG is adjacent to the perisylvian language processing network and is associated with the verbal processing of numbers (Dehaene et al., 2003). It is more highly activated during addition and multiplication than during subtraction, presumably because addition and multiplication facts are more likely to be retrieved from long-term memory (Stanescu-Cosson et al., 2000; Simon et al., 2002; Delazer et al., 2005). It is also more active during symbolic number processing than non-symbolic number processing (Holloway et al., 2010; Price and Ansari, 2011). Although the left AG is usually associated with verbal strategies for solving number processing problems, that is not likely to be the case here, as these non-symbolic number comparison problems do not involve the recall of arithmetic facts (Menon et al., 2000), verbal manipulations of number (Dehaene et al., 2003), or the mapping from symbols to numerical magnitudes (Grabner et al., 2007; Ansari, 2008). However, the left AG is also involved in visuospatial attention (Nobre et al., 1997; Corbetta and Shulman, 1998; Gitelman et al., 1999; Corbetta et al., 2000; Rushworth M.F.S. et al., 2001; Rushworth M.F. et al., 2001) and has been shown to be involved in mentally maintaining a spatial representation of numbers similar to a mental number line (Göbel et al., 2001). Based on our data, it appears that the exposed children rely on this spatial representation increasingly as the difficulty of the problems increases, instead of relying on quantity processing mediated by the right IPS.

### Effects on the PSPL

Exposed children activated the right PSPL less than control children, and more frequent drinking was associated with reduced activation. Although this effect was no longer significant after adjustment for maternal smoking during pregnancy, smoking was confounded with prenatal alcohol exposure in this sample (*r* = 0.44, *p* = 0.010), making it difficult to tease out the degree to which each of these exposures was implicated in the observed effect. Prenatal alcohol exposure has not been shown previously to affect the activation of the right PSPL during number processing, but in our previous study we found that greater prenatal alcohol exposure was related to less activation of the *left* PSPL during EA (Woods et al., 2015). Similarly, a study of subtraction (Santhanam et al., 2009) found that a similar region in the left hemisphere was activated more by controls than by exposed young adults with alcohol related dysmorphology. The PSPL, which is activated during counting (Piazza et al., 2002) and a variety of visual–spatial tasks, is believed to support the engagement of attention during visual processing of numbers (Pinel et al., 2001; Dehaene et al., 2003). These findings suggest that at this age alcohol exposed children seem to be less able to recruit the attentional systems associated with number processing.

Interestingly, activation patterns in the right PSPL for the non-syndromal HE children were similar to those in the FAS/PFAS group, while those in the right IPS were similar to controls. These data suggest that, although the activation of right PSPL appears to be impaired in the non-syndromal HE children, the functioning of the right IPS is apparently spared. Other studies have also found more extensive neural impairment in the FAS/PFAS group than in the HE. For example, both functional and structural connectivity have been found to be lower in heavily alcohol exposed children in only a subset of the regions affected in children with FAS/PFAS (Fan et al., 2016, 2017).

Greater activation of the left PSPL, as well as a greater distance effect on the left PSPL, were both associated with quicker and more accurate behavioral performance. Dehaene et al. (2003) have emphasized that the PSPL can be engaged when attending to specific quantities on the number line. It is possible that the children who are better able to recruit the left PSPL (and recruit it more with increasing task difficulty) are better able to position each array of stimuli on the mental number line and, therefore, to make magnitude comparisons more quickly and accurately.

### Comparisons of Effects on Symbolic and Non-symbolic Number Processing

Because our previous study (Woods et al., 2015) investigating the effect of prenatal alcohol exposure on symbolic number processing in children used the same *a priori* ROIs as in this study, the results of these two studies can be examined to compare the effect of prenatal alcohol exposure on symbolic and non-symbolic number processing. The tasks investigated in the previous study of 49 children (8–12 years of age) were simple EA and a magnitude comparison task, PJ.

In both studies, although the activation of the right IPS was impaired by prenatal alcohol exposure, we found no relation of alcohol or FASD diagnostic group to activation of the left IPS. It is not clear whether the right IPS is more affected by prenatal alcohol exposure than the left IPS or whether they are equally affected, but only the effect on the right IPS is apparent because the left IPS is recruited to a lesser extent by these tasks. In the Dehaene meta-analysis (Dehaene et al., 2003) used to identify the critical parietal number processing regions used in our studies, the IPS activation was bilateral in all but two studies. To examine whether the absence of an alcohol effect on the left may be due to differences in degree of activation on the left and right, we compared the magnitudes of the activation in the left and right IPS in control children during symbolic and non-symbolic number processing. In control children, neither the left nor the right IPS showed significant activation increases during non-symbolic number processing. In the PJ task, activation increases were seen bilaterally in the IPS (right IPS mean % signal change ± SD = 0.09 ± 0.12, *p* = 0.007; and left IPS mean % signal change ± SD = 0.07 ± 0.13, *p* = 0.045), but during EA activation increases were seen only on the right (right IPS mean % signal change ± SD = 0.07 ± 0.08, *p* = 0.003). It is, thus, not clear whether the left IPS is more sensitive to prenatal alcohol exposure or merely less active in the tasks examined to date.

While during the Smarties and PJ tasks, exposed children showed increased activation of the left AG, no compensatory activation was seen during EA (see also Meintjes et al., 2010). These data suggest that exposed children show compensatory activation of the left AG during magnitude comparison tasks, both symbolic and non-symbolic, but not during arithmetic tasks relying on verbal recall. Whereas for magnitude comparison tasks, the control children appear to use the optimal strategy, relying on the core quantity system in the IPS, the alcohol exposed children recruit the left AG to a greater extent, possibly due to alcohol-related damage to the IPS. In contrast, for arithmetic tasks, verbal recall of number facts (relying on the left AG) is the most efficient strategy and appears to be used by both the exposed and control children.

While prenatal alcohol exposure was associated with lower activation of the right PSPL during the Smarties task, no association with alcohol exposure was found in this region during either PJ or EA (see also Santhanam et al., 2009; Meintjes et al., 2010). In contrast, alcohol-related reductions were seen in activation in the left PSPL during EA (Woods et al., 2015) and in a comparable region during subtraction (Santhanam et al., 2009). Thus, it appears that both the left and right PSPL are impaired by prenatal alcohol exposure, but the impairment of the right PSPL is evident only during non-symbolic magnitude comparison, whereas impairment of the left PSPL has been seen during symbolic arithmetic operations. Neither of the studies using the PJ task to assess symbolic magnitude comparison found alcohol-related impairment in the left nor the right PSPL.

Although all but one of the heavily alcohol exposed children showed relatively low levels of activation in the right PSPL during the Smarties task, there was a notably wide range of signal change among the controls (see **Figure 4**). We examined numerous sample characteristics (child IQ, age at scan, grade in school, ADHD status, maternal education and SES, smoking and other prenatal drug exposures, and postnatal lead exposure) in relation to these activation levels in the control group. One of the two controls with the lowest % signal change had a somewhat elevated prenatal exposure to smoking (seven cigarettes/day). There were no other differences that would help explain the wide range in responses, suggesting that these activations represent a “normal” range for the controls. What is striking in our findings is that the activation levels in this brain region are low for virtually all of the alcohol-exposed children.

### Limitations

One limitation of this study was that, by contrast to most of the studies our group has conducted in Cape Town, the maternal report of drinking during pregnancy for this cohort was obtained retrospectively several years after the child’s birth. Nevertheless, the validity of these reports is supported by the fact that they were predictive of neuroimaging and neurobehavioral outcomes (Meintjes et al., 2010, 2014; Jacobson et al., 2011b,c; De Guio et al., 2014; du Plessis et al., 2014; Lewis et al., 2015; Lindinger et al., 2016). Predictive validity of the maternal pregnancy drinking reports for childhood IQ in this sample was substantial (*r*s = –0.54 and –0.53, for AA/day and AA/occasion, respectively, both *p*s < 0.001). However, drinking during pregnancy is more difficult to recall reliably in a retrospective interview than smoking, which is a more addictive, more consistent, and less of an episodic practice, as well as packaged so that quantity is more readily recalled (Jacobson and Jacobson, 1992). These differences in ability to recall quantity between alcohol exposure and smoking make it particularly difficult to tease out the degree to which each of these exposures is responsible for the outcomes, such as activation of the right PSPL, that are related to both exposures in this sample. A second limitation was the small size of the FAS/PFAS (*n* = 6) and HE (*n* = 5) groups. In addition, because all of the children come from socioeconomically and educationally disadvantaged environments, we cannot determine the degree to which the results would hold for children from an educationally less deprived background. We did not control for multiple comparisons, due to the fact that we examined only five regions rather than the whole brain; ROI analyses increase SNR by averaging across the voxels in a region. Although the use of an ROI approach was appropriate for this study, a whole brain voxelwise analysis could have revealed additional activation differences between groups.

## Conclusions

This study found poorer recruitment of the right IPS during non-symbolic number comparison in syndromal children with FAS/PFAS compared with controls, extending our previous finding of poorer right IPS recruitment during symbolic number processing (Woods et al., 2015). To our knowledge, this is the first to show that heavy prenatal alcohol exposure impairs mental representation and manipulation of quantity for non-symbolic, as well as symbolic, representations. As hypothesized, this impairment appeared to be compensated for by increased activation in the left AG, with only the exposed children recruiting the left AG to a greater extent as task difficulty increased. The inverse relation between prenatal alcohol exposure and activation of the right PSPL in children with prenatal alcohol exposure suggests that alcohol impairs the ability of exposed children to employ the attentional systems required for optimal number processing. Notably, the non-syndromal HE children’s activation was impaired in the right PSPL, which mediates attention during number processing, but spared in the right IPS, which mediates quantity comparison.

## Ethics Statement

Written informed consent was obtained from each mother and assent from each child. This study was conducted according to the ethical guidelines and principles of the international Declaration of Helsinki, as well as South African research ethics guidelines, as documented by the Department of Health in “Ethics in Health Research: Principles, Processes and Structures”, Second edition, 2015. Approval for human research was obtained from the Wayne State University Institutional Review Board and the UCT Faculty of Health Sciences Ethics Committee.

## Author Contributions

KW performed the neurobehavioral and neuroimaging data analyses, reviewed the literature, interpreted the findings, and wrote up the paper. EM provided overall project supervision, collaborated on the design of the neuroimaging study, oversaw the neuroimaging assessments, and reviewed the paper. SJ and JJ designed the original study and the neuroimaging task, supervised recruitment and maternal and child assessments, provided suggestions regarding data analysis and interpretation, and reviewed the paper. CM administered the maternal interviews, which included sociodemographic information and alcohol, smoking and drug ascertainment.

## Conflict of Interest Statement

The authors declare that the research was conducted in the absence of any commercial or financial relationships that could be construed as a potential conflict of interest. The handling editor VAR declared a shared affiliation, though no other collaboration, with the authors.
